# Perfusion culture maintained with an air-liquid interface to stimulate epithelial cell organization in renal organoids in vitro

**DOI:** 10.1186/s42490-019-0017-9

**Published:** 2019-07-23

**Authors:** Sachiko Sekiya, Tetsutaro Kikuchi, Tatsuya Shimizu

**Affiliations:** Institute of Advanced Biomedical and Engineering Science, Tokyo Women’s Medical University, Kawada-cho 8-1 TWIns, Shinjuku-ku, Tokyo, 162-8666 Japan

**Keywords:** Renal organoid, Perfusion culture, Air-liquid interface

## Abstract

**Background:**

Organoids derived from induced pluripotent stem (iPS) or embryonic stem (ES) cells have been evaluated as in vitro models of development and disease. However, maintaining these cells under long-term static culture conditions is difficult because of nutrition shortages and waste accumulation. To overcome these issues, perfusion culture systems are required for organoid technology. A system with a stable microenvironment, nutrient availability, and waste removal will accelerate organoid generation. The aim of this study was to develop a novel perfusion system for renal organoids by maintaining the air-liquid interface with a device fabricated using a 3D printer.

**Results:**

Our results revealed slow flow at the organoid cultivation area based on microbead movement on the membrane, which depended on the perfusion rate under the membrane. Moreover, the perfused culture medium below the organoids via a porous membrane diffused throughout the organoids, maintaining the air-liquid interface. The diffusion rates within organoids were increased according to the flow rate of the culture medium under the membrane. The perfused culture medium also stimulated cytoskeletal and basement membrane re-organization associated with promotion tubular formation under 2.5 μL/min flow culture. In contrast, tubules in organoids were diminished at a flow rate of 10 μL/min.

**Conclusions:**

Our liquid-air interface perfusion system accelerated organization of the renal organoids. These results suggest that suitable perfusion conditions can accelerate organization of epithelial cells and tissues in renal organoids in vitro.

**Electronic supplementary material:**

The online version of this article (10.1186/s42490-019-0017-9) contains supplementary material, which is available to authorized users.

## Background

Establishment of embryonic stem (ES) and inducible pluripotent stem (iPS) cell culture technologies allow for the generation of various cell types by in vitro differentiation. Moreover, three-dimensional (3D) culture of differentiated cells accelerates self-organization and biological functions better than traditional two-dimensional culture. Previously, “organoids” simply represented 3D cell-aggregates containing epithelial cysts or tubules [1]. Currently, organoids developed from ES or iPS cells have been evaluated as in vitro reproducible models of complex in vivo human tissues [2]. The retina, liver, lung, inner ear, etc. have already been generated as organoids from ES or iPS cells [3–6].

Renal organoids were produced in a study of renal progenitor differentiation [7–10] and contained metanephric mesenchymal cells and ureteric bud cells which organized into embryonic renal tubules in vitro. One of the differentiation protocols used to imitate the embryonic status was reported as an adaptation of 3D organotypic cultivation at the air-liquid interface [10]. They obtained much of the nephron structure with an endothelial cell network structure in vitro using iPS cells.

For continuous and progressive development, 3D organoids require a supply or perfusion of fresh culture media nutrients and the elimination of waste products. Similar to engineered 3D myocardial tissue or 3D hepatic structure [11, 12]*,* renal organoids require vascularization by co-cultivation with endothelial cells [13]. Particularly, the kidney has high nutrient and oxygen demands, similar to the heart, because of its specific function [14]. For example, proximal renal tubular epithelial cells are involved in transporting glucose, minerals, and water from the tubular environment to the external vasculature via transporters. Moreover, podocytes also produce a size-specific slit membrane to eliminate wastes from the blood by glomerular filtration. In fact, it was reported that cellular mitochondrial damage was associated with dysfunction of the podocytes and proximal tubules [15].

In addition to fresh medium supplementation, biomimetic flow stimulation is important for the maturation of epithelial polarity in 3D tissues. Because renal epithelial cells are always exposed to urinal flow on one side and abundant blood flow at the basolateral side, it is difficult to maintain their function under static culture conditions in vitro [16]. Therefore, several renal epithelial cell perfusion culture systems have been developed [17–19]. As a general method, monolayer epithelial cells are cultured on a porous membrane perfused on the apical side to imitate renal tubule flow [20]. Another perfusion system was fabricated to imitate the glomerular microenvironment by perfusion of co-cultured podocytes and endothelial cells via porous membranes [21]. A more accurate renal tubule structure was fabricated using a 3D bio-printer and perfused to examine the toxicity towards tubular cells in vitro. However, few studies have examined perfusion of renal organoids in vitro.

This study is the first to report a perfusion system with an air-liquid interface for the organotypic cultivation of renal organoids produced from human iPS cells. Because renal organoid structures cannot be maintained under submerged conditions, our system was developed for medium perfusion under renal organoids on porous membranes to maintain an air-liquid interface. In the embryo, the kidney is perfused by an immature and leaky renal vasculature [22]. The low flow produced by our perfusion system may mimic the embryonic renal blood perfusion, providing adequate flow for renal organoid perfusion. This method can be used for long-term cultivation and maturation of organoids in vitro.

## Results

### Renal organoids were induced from hiPS cells

**Fig. 1:**
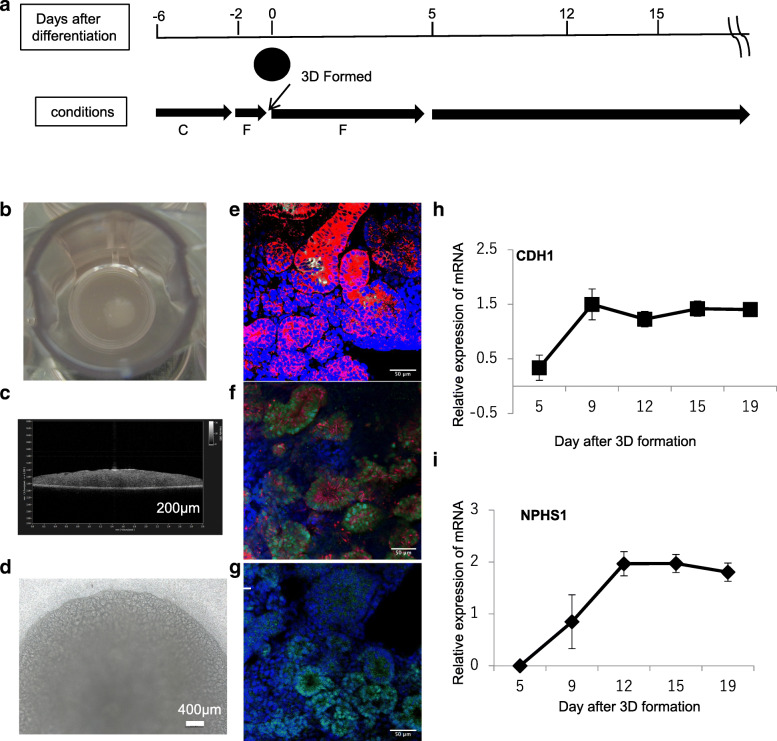
**a** Procedure of renal organoid production. C indicates CHIR-99021 and F indicates FGF9. The detailed protocol is described in the Methods section. **b** Macroscopic image. **c** OCT section image of organoids at 12 days after 3D formation. **d** Phase contrast microscopic image of a renal organoid **e**) immunostaining with E-cadherin (red fluorescence) and FITC-LET lectin (green fluorescence), or **f**) CK8 (red fluorescence) and PAX2 (green fluorescence) and **g**) WT1 (green fluorescence). Blue fluorescence indicates the counterstained cell nuclei (**e–g**). CDH1 (**h**) and NPHS1 (**i**) gene expression after 3D formation of renal organoid induction is indicated as graphs

The protocol for renal organoid production allowed us to obtain epithelial cells and determine nephrin expression in proximal epithelial cells in 3D organoids until 12 days after 3D structure formation (Fig. 1a). Ellipsoidal organoids more than approximately 5 mm in diameter and 400 μm thick were obtained on a cell culture insert membrane (Fig. 1b,c) at 12 days after 3D formation. These organoids showed a well-developed tubular structure, as confirmed by phase-contrast microscopy (Fig. 1d). E-Cadherin immunostaining and *Lotus tetragonolobus* lectin (LTL) staining results revealed proximal tubules (PT) as E-cadherin, with LTL-positive tubules, distal tubules (DT), and ureteric bud cells stained as E-cadherin-positive and LTL-negative tubules (Fig. 1e). At 15 days after organoid formation, EMX2, SIM1, and GATA3, ureteric bud cells markers, were also expressed in the organoid (Additional file 1) The results indicate that organoids at day 12 after 3D formation contained PT and DT and included ureteric bud cells. Figure 1 f shows the results of cytokeratin 8 (CK8) and PAX2 immunostaining. CK8-positive cells were similar to E-cadherin- and also PAX2-positive cells. PAX2 is a transcription factor for mesenchymal-to epithelial transition in renal development. WT1 is also a transcription factor that functions in nephron development. WT1-positive cells were also observed in these tubules. Thus, renal development occurred in day12 organoids after 3D formation. Furthermore, gene expression of CDH1 (E-cadherin), an epithelial cell marker, and NPHS1 (nephrin 1), a podocyte marker, was increased at day 9 after 3D formation. The expression of these genes plateaued until 19 days after 3D formation. Thus, renal organoids containing renal tubules were obtained until 12 days after 3D formation from hiPS cells.

### Culture medium perfusion of renal organoids with a device maintaining the air-liquid interface

**Fig. 2:**
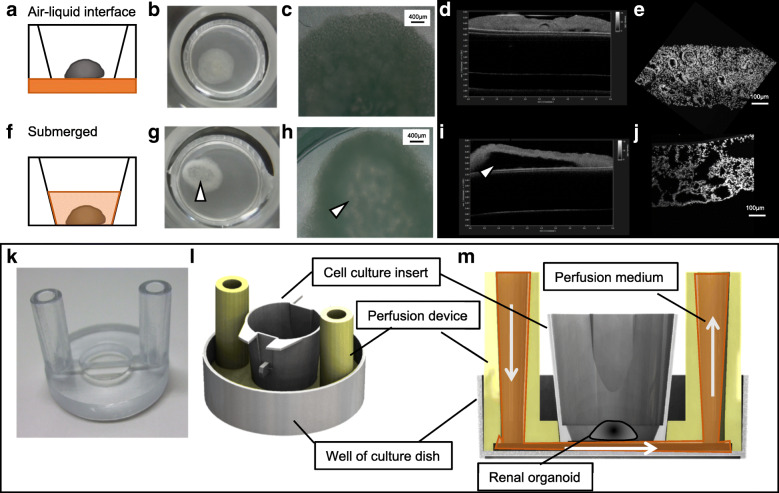
Organoids cultured at the air-liquid interface condition (**a–e**) or submerged (**f–j**) at 12 days after 3D formation. **a** and **f** indicate the schematic of the condition and real macroscopy. **b,g**, microscopic (phase contrast) images (**c,h**) at 3 days (15 days after 3D formation) after cultivation. **d** and **i** indicate the OTC images and nuclei-stained cryo-sections of these organoids (**e, j**). **k** White arrow heads at g)~i) were indicated degradation structure of organoid. The original device fabricated using the 3D printer (**k**). Schematic explaining the cell culture insert setting (**l**) and flowing perfusion medium under the organoid on the porous membrane in cell culture inserts (**m**)

Renal organoids induced by organotypic cultivation could not maintain their structures for 3 days after switching to submerged culture conditions (Fig. 2a,f). Compared to organotypic cultivation, the bottom of the organoid was decayed under the submerged condition (Fig. 2b–d and g–i). The tubular structure in submerged cultured organoids was diminished (Fig. 2e and j), indicating that the air-liquid interface was important for the continuous growth of organoids. Therefore, we fabricated a device for renal organoid perfusion using a 3D printer to maintain the air-liquid interface on the porous membrane (Fig. 2k). This device supported the cell culture inserts and was connected with micropumps for perfusion (Fig. 2l, m). The flow of the perfusion medium was maintained at the first inlet in the chamber under porous membranes supporting organoids. Because of this, the device could replace the medium and prevent stress stimulation in the cultured organoids.

### Simulation of flux on the membrane during perfusion under the membrane

**Fig. 3:**
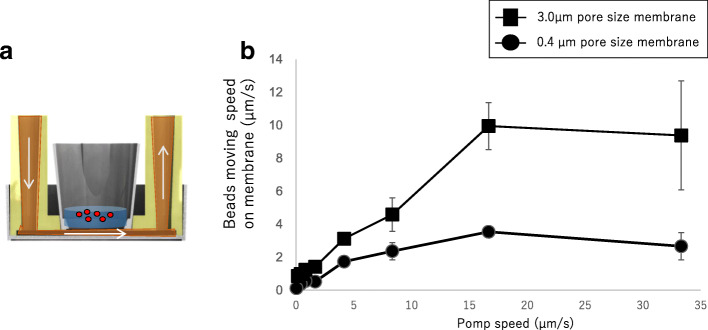
Analysis of microbead movement on the membrane. **a** Beads movement tracing experiment. **b** Bead movement speed alteration depend on pump perfusion volume and membrane pore size

To demonstrate flux on the membrane, the movement of fluorescence beads in the suspension on the membrane during perfusion was traced by time-lapse microscopy. Fluorescent beads exhibited turbulent flow on the membrane (movement movie shown in Additional file 2) and movement according to the flow volume under the membrane. This movement was increased by increasing the pore size in the membrane from 0.4 to 3.0 μm (Fig. 3). These results indicate that perfusion under the membrane results in flux on the membrane during perfusion.

### Tracing perfused medium diffusion in the renal organoids

**Fig. 4:**
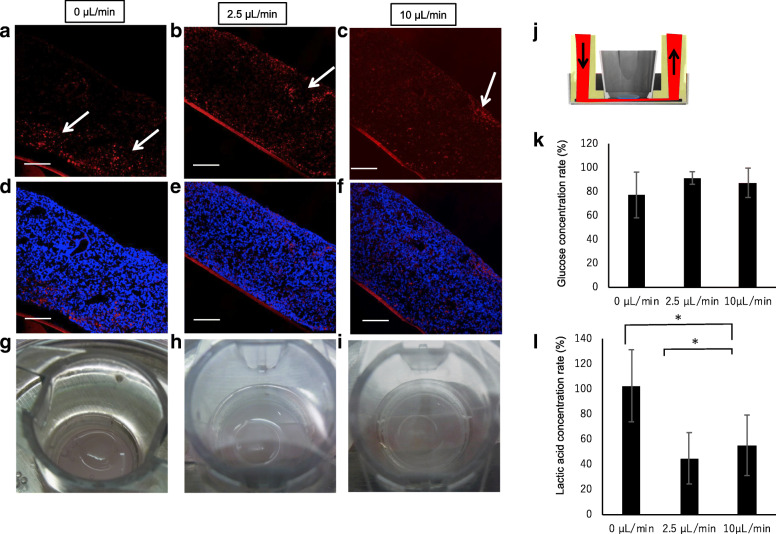
Perfused medium tracing with Texas Red-labeled dextran in the organoid. **a–f** sections of 0 μL/min (static condition) (**a, d**)**,** 2.5 μL/min perfusion (**b, e**), and 10 μL/min perfusion (**c, f**) conditions. **a–c** indicate Texas Red-labeled dextran-positive images and **d–f**) indicate Texas Red-labeled dextran and nuclear staining merged images. **g–i** Macroscopic images of individual conditions confirming the maintained air-liquid interface. **j** Illustration of the medium perfusion tracing experiment, **k** and **l** indicate the relative concentration of glucose and lactic acid in the culture medium after 48 h of incubation using sham condition medium as a standard. Sham was incubated in a medium without organoid culture for 48 h

To clarify the diffusion of perfused medium within the organoids, renal organoids were perfused with medium containing Texas Red-conjugated dextran for 2 days. The organoids were then examined for diffusion at different depths by cryo-sectioning after fixation. Dextran-positive sections showed non-specific attachment or endocytosis associated with micropinocytosis due to diffusion of the culture medium in the organoids during perfusion culture. Even under non-perfusion conditions, Texas Red-conjugated dextran was slightly percolated into the renal organoids around the porous membrane (Fig. 4a, d). Interestingly, the culture medium under the organoids diffused throughout the renal organoids to a greater extent at a flow rate of 2.5 or 10 μL/min than under the static condition (Fig. 4b–f), despite maintaining the air-liquid interface during incubation in all groups (Fig. 4g–i). Consumption of glucose in the medium and production of lactic acid during cultivation are shown in Fig. 4g. The glucose concentration in the medium after 48 h was similar under both conditions, whereas the lactic acid concentration was altered by the perfusion conditions. Compared to under static conditions, the lactic acid concentration was decreased under the 2.5 and 10 μL/min conditions. These results indicate that perfusion culture can accelerate medium diffusion throughout the renal organoids, despite maintaining an air-liquid interface condition. Additionally, the perfusion culture altered organoid metabolism in the culture system.

### Perfusion medium diffusion increased with flow rate within the renal organoids

**Fig. 5:**
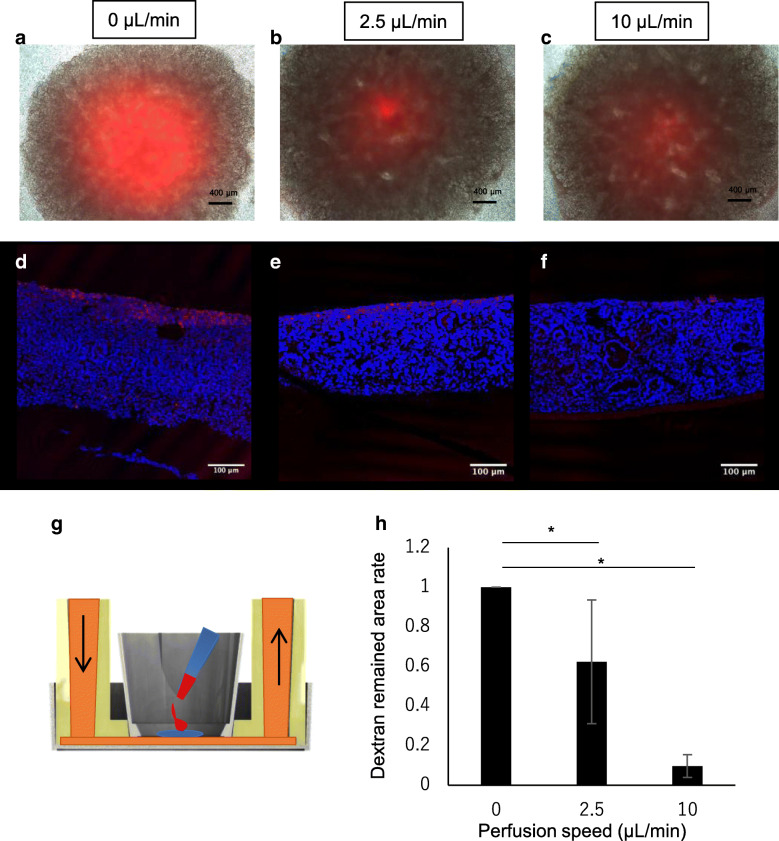
Dextran labeled with Texas Red dropped on the organoid cultivated for 2 days under static or perfusion conditions. **a**–**c** Microscopic merged images of Texas Red fluorescence and phase contrast images. Red color indicates the remaining Texas Red-labeled dextran. **d**–**f** Texas Red-labeled dextran and nuclear staining merged images. **g** Schematic explaining the dropped dextran at the start of cultivation. **h** Summary remaining dextran in the sections from (**d–f**) (*n* = 3)

Upon addition of 0.5 μL of 1 mg/mL Texas Red-conjugated dextran at the top of the organoids, the dextran immediately diffused following the movement of water in the organoid. Therefore, we quantitatively analyzed the movement of water in the organoids in which Texas Red-conjugated dextran was added. The remaining dextran-positive areas in the organoid sections were calculated and shown as a graph (Fig. 5h). Compared to the static condition, the perfused condition showed diffusion of Texas Red-conjugated dextran (Fig. 5a–f). These results indicate that the diffusion rate throughout the renal organoid at the air-liquid interface increased according to the flow rate.

### Cytoskeletal re-organization by perfusion culture

**Fig. 6:**
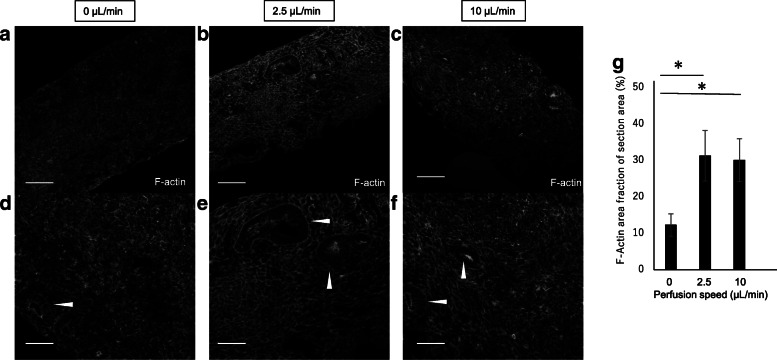
Actin staining after renal organoid sections were cultured under 0 mL/min **a,d**), 2.5 mL/min **b,e**), and 10 μL/min **c,f**) perfusion conditions for 2 days

The cytoskeleton of renal epithelial cells is known to react to shear stresses. To confirm the cytoskeletal alteration by perfusion culture, we examined the cytoskeletal morphology between static and perfusion conditions by F-actin staining. Indeed, the cytoskeleton in the perfusion conditions showed re-organization compared to that under static conditions (Fig. 6). F-Actin expression in the renal epithelial cells was strong under both perfusion culture conditions. Moreover, the basement point of the cytoskeletal structure was clear in the 2.5 μL/min flow condition compared to that in the 10 μL/min flow condition. By quantification of the F-actin structure in the section, the positive area increased in the perfusion organoid sections (Fig. 6h). These results implied that renal organoids may be exposed to a small flow because of the perfusion conditions.

### Perfusion medium stimulated epithelial tubular formation in renal organoids

**Fig. 7:**
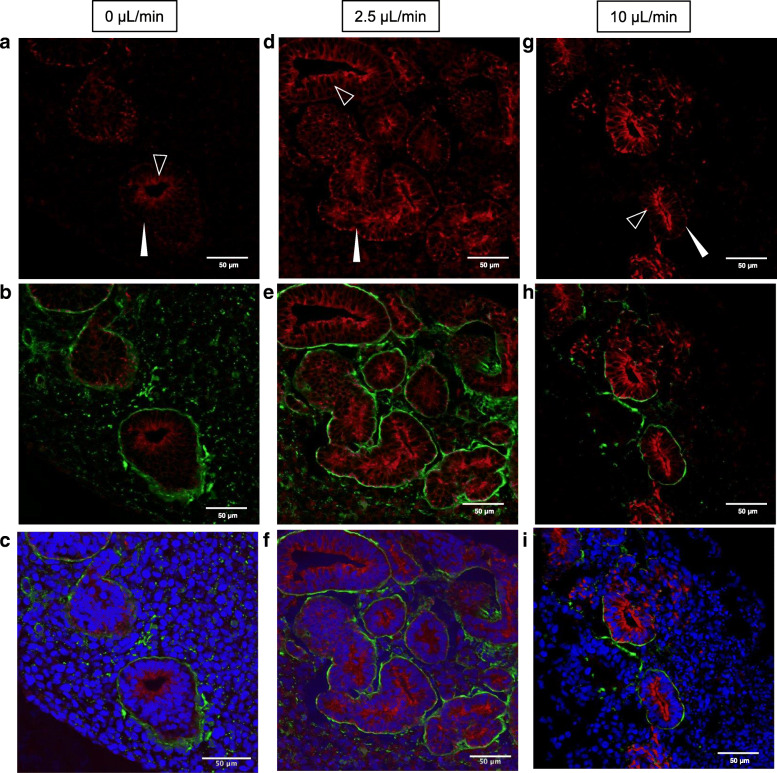
CK8 and laminin immunostaining of organoid sections after 2 days under all culture conditions. **a, d**, and **g** CK8-stained images. **b, e**, and **h** CK8- (red) and laminin-stained (green) merged images. **c, f**, and **i** CK8 (red), laminin (green), and cell nuclei (blue) merged images

Because the cytoskeleton of epithelial cells is associated with hemidesmosome structures in the basement membrane and cytokeratin, these structures were examined by immunostaining of the renal organoids using laminin and CK 8 antibodies. Under static conditions, non-uniform laminin expression was observed around the epithelial tubules in renal organoids (Fig. 7 b). CK 8 expression was also easily detectable at the apical (inner) side of the tubules (Fig. 7a). In contrast, nearly uniform and continuous expression of laminin was observed around the epithelial tubules under the 2.5 μL/min flow condition (Fig. 7e). CK 8 was clearly observed at both apical sides of the tubules under both perfusion conditions. Under the 10 μL/min flow condition, a laminin-positive region was formed around the epithelial tubules and was non-continuous compared to that under the 2.5 μL/min condition (Fig. 6h). Moreover, CK 8 expression at the basolateral side of the tubules was non-continuous under the 10 μL/min perfusion condition. These immunostaining results suggest that the perfusion medium stimulated basement membrane and cytokeratin re-organization based on the perfusion volume.

**Fig. 8:**
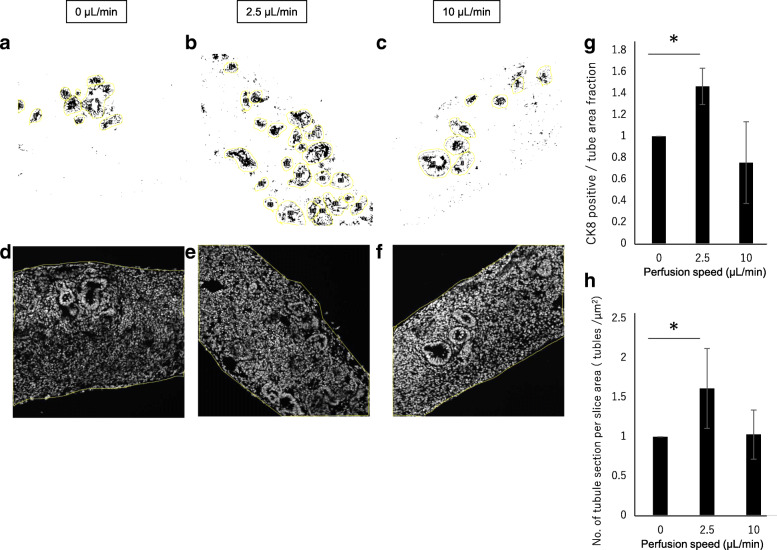
Quantification of CK8 immunostaining of renal organoid sections. **a–c** Representative tubule sections (yellow) under each perfusion condition by Fiji. CK8-positive area fraction in the tubule selected area was measured and summarized in graph g). **d–f** Representative whole section area of the tubule counted section under each perfusion condition. The summarized tubule counting results in the graph h)

To quantity CK8-positive epithelial tubular formation, the CK8-positive area in the tubule section area was calculated from each image (Fig. 8 a–f). CK8 was an intermediate filament in epithelial cells and associated with the epithelial cytoskeleton. The CK8-positive area was increased at the 2.5 μL/min perfusion rate (Fig. 8g). However, at the 10 μL/min perfusion rate, CK8-positive tubules in the section were not stable. Moreover, the number of tubule sections in the whole section area was increased at the 2.5 μL/min perfusion rate (Fig. 8h).

These results indicate that the air-liquid perfusion culture system affected tubular organization in the renal organoids.

## Discussion

In summary, a perfusion system maintained by an air-liquid interface was developed to culture renal organoids induced from hiPS cells (Figs. 1 and 2). The system perfused culture medium under the membrane which lifted the organoid, resulting in low flux on the membrane (Fig. 3) throughout the renal organoids while maintaining the air-liquid interface (Fig. 4). Diffusion within the organoid increased according to the flow rate (Fig. 5). The perfusion condition may affect the microenvironment in renal organoids and alter the cytoskeleton and basement membrane (Fig. 7). This was correlated with an altered frequency of epithelial tubules in the renal organoid by the perfusion conditions (Fig. 8).

Switching to submerged conditions may affect the established epithelial cell polarity in renal organoids at 12 days after 3D formation under an air-liquid interface condition [23], and thus the tubule structure in the renal organoids could not be maintained under submerged conditions. To overcome this issue, our perfusion system was used to perfuse culture medium under the renal organoid while maintaining an air-liquid interface. An increasing perfusion rate accelerated flux on the membrane and diffusion medium in the organoid. Because water pressure in the flow channel was higher than that in the outlet channel, the medium flowed continuously. This may cause vertical direction flow over and small flux on the porous membrane. Alternatively, the perfusion conditions may lead to increased cell mobility compared to under static conditions. Indeed, because ureteric bud cell branching and mesenchymal-epithelial transition occurred in the renal organoids, the number of newly formed tubules increased in the organoids under the perfusion condition (Fig. 8). Thus, perfusion culture stimulated cell movement in the renal organoid, and diffused culture medium in the organoid was increased according to the perfusion condition.

Renal epithelial cells cultured under perfusion conditions at the apical side exhibited cytoskeletal reorganization at 1 dyn/cm^2^ shear stress. In endothelial cells, this re-organization was induced by over 10 dyn/cm^2^ shear stress. Thus, renal epithelial cells are more sensitive to perfusion stimulation than endothelial cells [18]. However, 0.5 Pa (mathematically 5 dyn/cm^2^) shear stress was also reported to cause cytoskeletal disruption in renal proximal epithelial cells [24]. Based on previous studies and our results, an adequate flow rate is required to maintain the structure of renal proximal tubular cells. Particularly, in the metanephros, ureteric buds increased branching with membrane-type matrix metalloproteinase (MT-MMP), MMP2, and MMP9 expression in the metanephric mesenchyme [25]. These enzymes digest the basement membrane and allow for elongation of branches. Secreted factors in the metanephros include MMP as well as fibroblast growth factor 2 (FGF2) and glial cell-derived neurotrophic factor [26]. These proteins diffused in the organoid via the perfusion medium. Diffusion of growth factors may reduce their effects on target cells, and thus may reduce tubules in the 10 μL/min flow rate condition compared to in the 2.5 μL/min flow rate condition. The epithelial cell basement containing laminin may possibly break at several points under the 10 μL/min flow rate condition (Fig. 7g–i).

In renal development, the metanephros receives blood perfusion from embryonic circulation. The metanephric vasculature is reported to show high permeability such that the perfused blood is leaked into the metanephros stroma [22]. Nephron progenitors exist at the edge of the metanephros, where they receive a low blood supply from embryonic blood circulation. Thus, excessive diffusion in the embryonic kidney may affect nephron progenitors in the metanephros. Maternal circulation can also affect embryonic circulation via the placenta. Maternal conditions such as hypertension, diabetes, and low nutrition may result in the development of low numbers of nephrons and are associated with future diseases in their offspring [27–29]. For example, maternal blood pressure alterations in rats cause hypertension in their offspring [30]. While animal experiments reveal important features of this phenomenon, species differences may also occur. Thus, renal organoids from hiPS cells cultured for a long time under perfusion culture simulating embryonic circulation may also improve the understanding of embryonic circulation effects on renal development. This perfusion system can be used to model embryonic renal development as well as renal diseases.

Recently, an interesting renal organoid perfusion system was reported [31] which demonstrated endothelial cell network perfusion in the renal organoid. However, the matrix gel was degraded and environment was altered by including cells during cultivation. Because our system without gel could be perfused at a constant rate for a long time, it may also be suitable for maintaining tissues such as the cornea and dermal skin tissue at the air-liquid interface. For mature organ engineering, gel-embed perfusion showed powerful effects as a cultivation system. To achieve functional renal tissue engineering, an adequate matrix gel will be applied in our culture system in the future.

## Conclusions

In this study, an air-liquid interface perfusion system was established for renal organoid in vitro cultivation. In this system, low flux on the porous membrane occurred via perfusion medium under the membrane within renal organoid. The diffusion also altered growth factor diffusion and renal tubule organization. This perfusion cultivation system will be improved to control the organization of tubule and vascularization in the renal organoid in vitro.

## Methods

### Cell culture and renal organoid production

Human iPS cells (201B7, Lot No. 018) were obtained from RIKEN BRC Cell Bank (Tukuba, Japan) [32] and cultured on mouse embryonic fibroblast feeder cells (ReproCELL, Inc., Kanagawa, Japan) in an incubator with 5% CO_2_. The cells were then cultured using standard feeder-less cultivation procedures. Briefly, the cells were plated on Laminin 511 (imatrix-511, Matrixome, Inc., Osaka, Japan)-coated culture dishes (35-mm diameter) and cultured with StemFit®AK02N (ReproCELL). iPS cells were stocked in liquid nitrogen and used for renal organoid induction from passage 10 to 21. Renal organoids were produced as previously reported with some modifications [10]. Briefly (as shown in Fig. 1 a), when undifferentiated hiPS cells were cultured to approximately 50% confluence on 35-mm diameter culture dishes, the culture medium was changed to renal organoid-induction medium (Stemdiff APEL2 medium, STEMCELL Technologies, Inc., Vancouver, British Columbia) containing 8 μM CHIR99021 (FUJIFILM Wako Pure Chemical Corporation, Osaka, Japan) and cultivated for 4 days. The medium was then changed to producing medium containing 200 ng/mL FGF9 and 1 μg/mL heparin and cultured for 2 days. The cultured cells were harvested with 0.5× TrypLE™ (Gibco, Grand Island, NY, USA) as a cell suspension. The suspended cells were collected in 1.5-mL tubes with 6.5 × 10^5^ cells/tube and centrifuged at 400×*g* for 2 min to form pellets. After centrifugation, the pellets were transferred onto cell culture inserts (Falcon™ cell culture insert, 0.4-μm pore size (1 × 10^8^ pores /cm^2^) for a 12-well plate, Corning Inc., NY). Renal organoid-induction medium containing 5 μM CHIR99021 and 10 nM Rock Inhibitor was added only under the cell culture insert and incubated with the pellets for 1 h. The medium was then replaced with renal organoid-induction medium containing 200 ng/mL FGF9 and 1 μg/mL heparin for 5 days. After 5 days of FGF9 treatment, the medium was replaced with renal organoid-induction medium without any factors. The culture medium was changed every 2 days and antibiotic agent (Antibiotic-Antimycotic, Gibco) was always added to the renal organoid-induction medium. The cultured pellets were grown as renal organoids until 12 days after pellet formation (3D formation). For submerged culture, the renal organoids were submerged in culture medium with the insert membrane in a 12-well plate. Phase-contrast images were captured with a phase-contrast microscope (Nikon Corporation, Tokyo, Japan) connected to an Axiocam controlled by Axio vision software (Carl Zeiss AG, Oberkochen, Germany).

### Microbeads movement experiment

To evaluate flow on the membrane in the culture system, we set up culture system without organoids but with approximately 20 μL red fluorescence 10-μm diameter polystyrene microbeads (3.6 × 10^6^ beads /mL, FluoSpheres™**,** Thermo Fisher Scientific, Waltham, MA, USA) suspended in 300 μL PBS on the membrane. Movement was captured by time-laps fluorescence microscopy every 2 s during 60–90 s perfusion at 37 °C. The pump speed (Icams lab) was changed to 2000, 1000, 500, 250, 100, 50, 25 μL/min. Movement series were captured twice at each speed. Time-lapse images were traced for 5 beads per image (over 30 images per series) with time-laps software (Aquacosmos 2.6, Hamamatsu Photonics K.K**.,** Shizuoka, Japan) and the bead movement speed (μm/s) was calculated.

### Measurement of glucose and lactic acid concentration in culture medium

At 48 h after cultivation, the medium directly under the organoid was collected and stored at − 80 °C. Glucose and lactic acid concentrations were examined by Oriental Yeast Co., Ltd. (Tokyo, Japan). We calculated the concentration rate both glucose and lactic acid using the value obtained from the sham experiment medium (incubation for 48 h in culture incubator) as a standard value (*n* = 7).

### Perfusion of renal organoids with the perfusion culture system

Perfusion devices supported by the cell culture insert were fabricated using a 3D-printer (EDEN; Object Geometries Billerica, MA, USA). The devices were placed into 6-well plates and connected to a microtube pump (Icams Lab Co. Ltd., Iwate, Japan) with TIGON Tubes. The complete perfusion system was placed in a CO_2_ incubator, and circulation of approximately 2.5 mL induction medium was maintained with the renal organoid at the air-liquid interface. As a control, support devices (kindly gifted by Dr. Itoga) fabricated using EDEN were used to adjust the total volume of the medium while maintaining the air-liquid interface. The medium for perfusion culture was changed every 2 days after cultivation.

### Tracing of diffused medium using fluorescence labeled dextran

To trace the perfusion of culture medium into the renal organoids, the medium was changed to medium containing 0.5 mg/mL Texas Red-conjugated dextran (70,000 MW Molecular Probes, Eugene, OR, USA). For 2 days of perfusion culture, the organoids were rinsed gently with PBS twice to remove the remaining medium labeled with dextran and fixed with 4% paraformaldehyde (Muto Pure Chemicals, Co. Ltd., Tokyo, Japan) for 24 h at 4 °C. To quantitatively analyze medium perfusion within the organoid, 0.5 μL of 1 mg/mL Texas Red-conjugated dextran was dropped onto the center of the top of the organoid using a micro-pipette. These organoids were cultured under static or perfusion conditions for 2 days and then fixed with 4% paraformaldehyde for 24 h at 4 °C. Cryoblocks were prepared with OTC Compound (Sakura Finetech Japan Co., Ltd., Tokyo, Japan). The blocks were sectioned using a cryostat at 8 μm thickness. The sections were dried and washed with PBS, and counterstained with 2 μg/mL Hoechst 33258 for 15 min. The sections were then washed, mounted, and analyzed by confocal laser microscopy with an FV1200 IX83 (Olympus, Tokyo, Japan). Texas red-positive areas of the sections were evaluated using Fiji software (*n* = 4; NIH, Bethesda, MD, USA).

### Immunohistochemistry

Cryosections of 2- or 3-day perfusion cultured renal organoids were washed with PBS and blocked with 0.1% bovine serum albumin/PBS (Sigma Aldrich, St. Louis, MO, USA) for 1 h. They were then incubated with Laminin (L9393, Sigma-Aldrich), CK8 (ab9023, Abcam, Cambridge UK), and podocalyxin (AF1658 R&D Systems, Minneapolis, MN, USA) antibodies at 4 °C. After incubation, the sections were washed with PBS and incubated with secondary antibodies (Jackson ImmunoResearch Laboratories, Inc., West Grove, PA, USA) for 2 h. The sections were washed with PBS, counterstained with 2 μg/mL Hoechst 33258 for 15 min, washed again, and mounted. Images were observed with a confocal microscope (Olympus).

### Quantification of images

Alexa 488-conjugated phalloidin-stained and CK8 immunostaining images captured at 200× with a confocal microscope. Three images per experiment were analyzed using Fiji software. Separated colors associated with each stain and tubule structure selected in the images were used to measure area fractions. The phalloidin (F-actin)-positive area in the whole section image and CK8-positive area fraction in the selected tubule area was calculated from the measurement. CK8-positive tubules in the section area were counted. All area fractions or frequencies are indicated as comparisons to the control value in each experiment (*n* = 3).

### Optical coherence tomography (OCT)

Three-dimensional images of the constructs were acquired by optical coherence tomography (OCT) (IVS-2000) (Santec Corporation, Aichi, Japan). The step size is indicated every 100 μm on the vertical axis and every 200 μm on the horizontal axis in the images (Figs. 1c, 2d, i).

### Gene expression

RNA in the organoids was extracted using a total RNA extraction kit (PureLink™ RNA mini kit, Thermo Fisher Scientific). cDNA was synthesized from 580 ng total RNA using a High-capacity cDNA reverse Transcription kit (Thermo Fisher Scientific). Real time PCR was performed using Taqman probes for CDH, NHPS1, SIM1, EMX2, and GATA3 on the Viia 7 real-time PCR system (Thermo Fisher Scientific). Beta-actin was used as an internal standard for NHPS1 and CDH1. Gene expression was quantified by the relative standard curve method (n = 3). For GATA3, SIM1, and EMX2, GAPDH was used for an internal standard gene. Gene expression was quantified by the ∆∆Ct method (day 12 *n* = 2, day 13 *n* = 1, day 15 *n* = 6). Calibration of these gene was conducted in iPS cells.

### Statistical analysis

The Tukey-Kramer HSD test was used to identify significant differences among multiple test groups by JMP Pro 14 (SAS Institute, Cary, NC, USA). All tests were two-tailed, and *P* < 0.05 was considered significant.

## Additional files

Additional file 1: Gene expression of EMX2, SIM1 and GATA3 in the induction renal organoid in static condition. Internal control was GAPDH. (PPTX 52 kb)

Additional file 2: Microbeads movement movie on the membrane during perfusion under the membrane. a) the illustration of the beads-movement tracing experiments. b) beads movement movie on 0.4 μm pore size membrane by 50 μL/min flow rate. c) beads movement movie on 3.0 μm pore size membrane by 50 μL/min flow rate. (PPTX 773 kb)

## Data Availability

Not applicable.

## Abbreviations

3D: Three-dimensional
CDH1: E-cadherin,
CK8: Cytokeratin 8
EMX2: Empty spiracles homeobox 2
ES: Embryonic stem
FGF2: Fibroblast growth factor 2
FITC: Fluorescein isothiocyanate
GATA3: GATA binding protein 3
GDNF: Glial cell-derived neurotrophic factor
iPS: Inducible pluripotent stem
LTL: *Lotus tetragonolobus* lectin
MT-MMP: Membrane-type matrix metalloproteinase
NPHS1: nephrin 1
SIM1: Single-minded homolog 1
