# Supplementary material for: Perfusion culture maintained with an air-liquid interface to stimulate epithelial cell organization in renal organoids in vitro
Source: BMC Biomed Eng. 2019 Jul 23;1:15. doi: 10.1186/s42490-019-0017-9 (PMC7422605; doi:10.1186/s42490-019-0017-9)
Supplement: Supplementary file 1 — Gene expression of EMX2, SIM1 and GATA3 in the induction renal organoid in static condition. Internal control was GAPDH. (PPTX 52 kb) [file 42490_2019_17_MOESM1_ESM.pptx]

## Slide 1
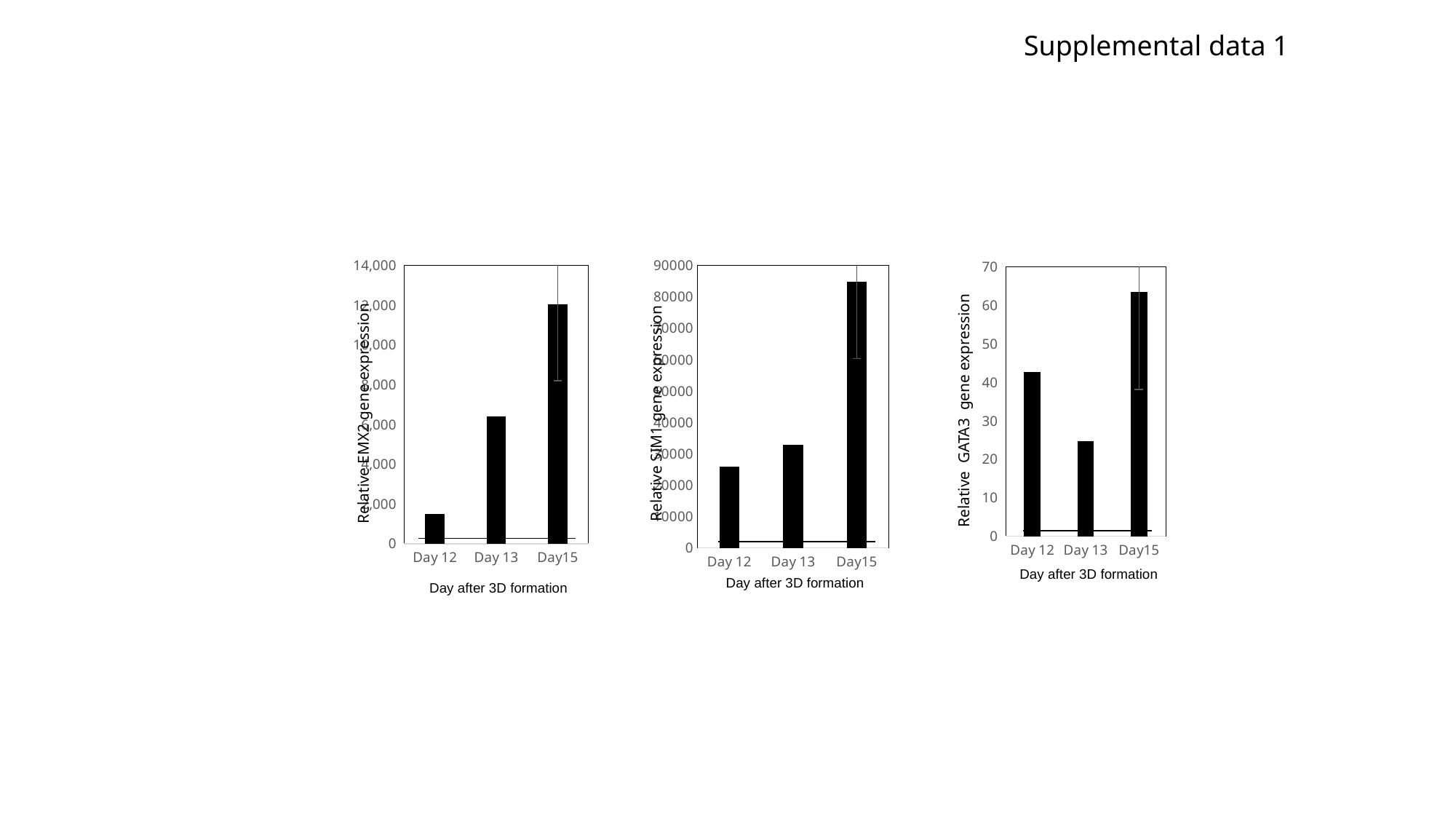

Supplemental data 1
### Chart
| Category | EMX2 |
|---|---|
| Day 12 | 1477.2904694725455 |
| Day 13 | 6402.444143632244 |
| Day15 | 12031.567205066618 |
### Chart
| Category | SIM1 |
|---|---|
| Day 12 | 25822.70857031353 |
| Day 13 | 32742.961092628673 |
| Day15 | 84832.24446017231 |
### Chart
| Category | GATA3 |
|---|---|
| Day 12 | 42.72435312352745 |
| Day 13 | 24.769773766413163 |
| Day15 | 63.549407250370024 |Relative GATA3 gene expression
Relative EMX2 gene expression
Relative SIM1 gene expression
Day after 3D formation
Day after 3D formation
Day after 3D formation
