# Supplementary figures and images for: Perfusion culture maintained with an air-liquid interface to stimulate epithelial cell organization in renal organoids in vitro
Source: BMC Biomed Eng. 2019 Jul 23;1:15. doi: 10.1186/s42490-019-0017-9 (PMC7422605; doi:10.1186/s42490-019-0017-9)

## Slide 1
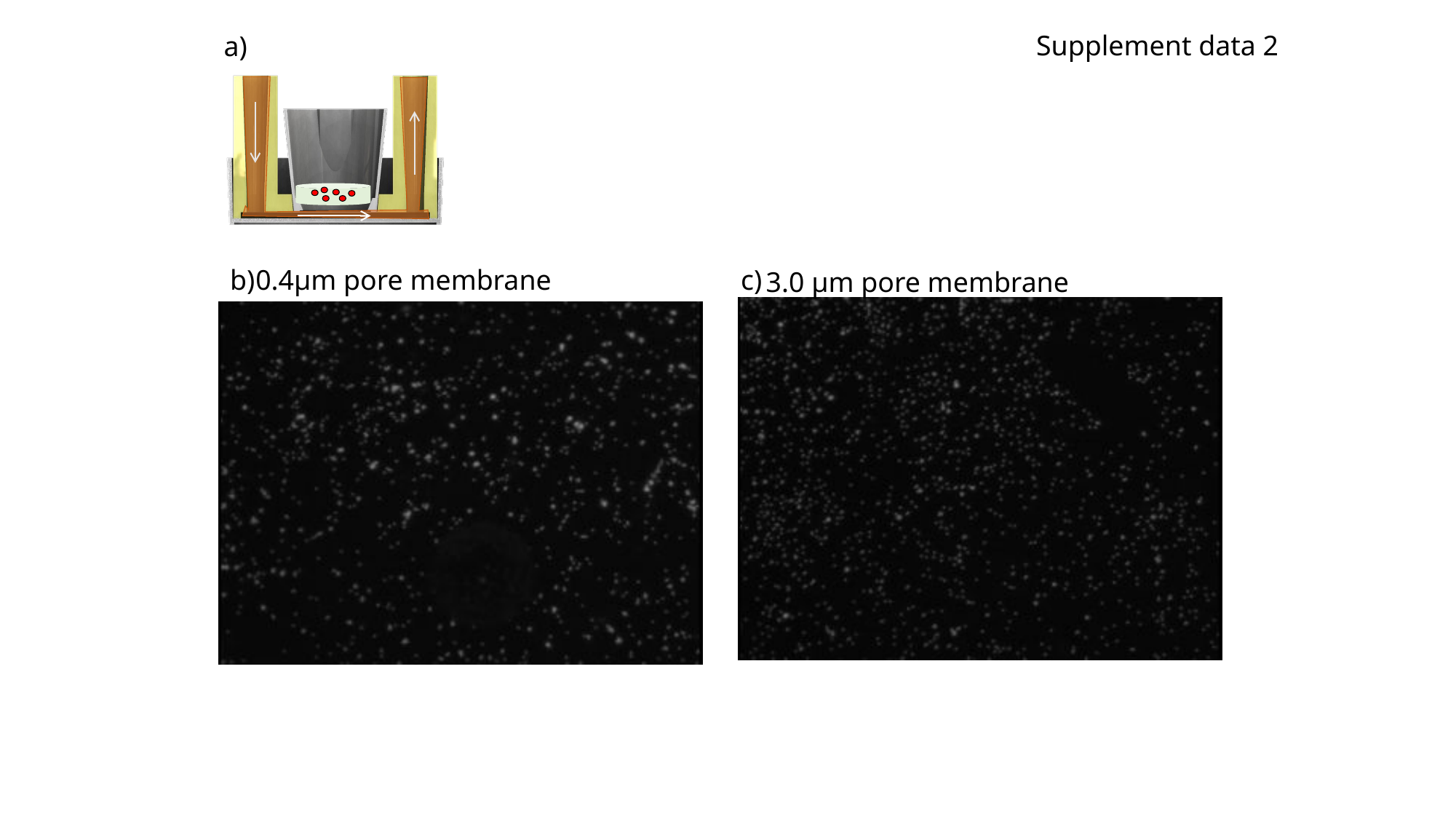

Supplement data 2
a)
b)
0.4µm pore membrane
c)
3.0 µm pore membrane

Supplement: Supplementary file 2 — Microbeads movement movie on the membrane during perfusion under the membrane. a) the illustration of the beads-movement tracing experiments. b) beads movement movie on 0.4 μm pore size membrane by 50 μL/min flow rate. c) beads movement movie on 3.0 μm pore size membrane by 50 μL/min flow rate. (PPTX 773 kb) [file 42490_2019_17_MOESM2_ESM.pptx]
